# Supplementary figures and images for: Show-through removal with sparsity-based blind deconvolution
Source: PLoS One. 2024 Jun 12;19(6):e0305208. doi: 10.1371/journal.pone.0305208 (PMC11168677; doi:10.1371/journal.pone.0305208)

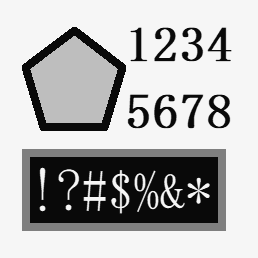

Supplement: S1 File — (ZIP) [file pone.0305208.s001.zip › S1_File/im_sim1_back.bmp]

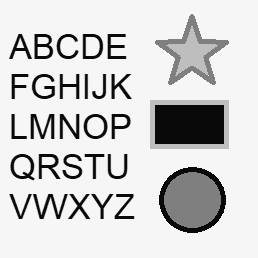

Supplement: S1 File — (ZIP) [file pone.0305208.s001.zip › S1_File/im_sim1_front.bmp]

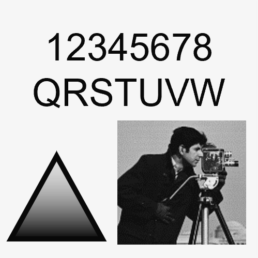

Supplement: S1 File — (ZIP) [file pone.0305208.s001.zip › S1_File/im_sim2_back.bmp]

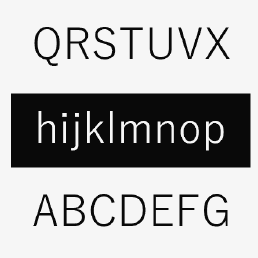

Supplement: S1 File — (ZIP) [file pone.0305208.s001.zip › S1_File/im_sim2_front.bmp]

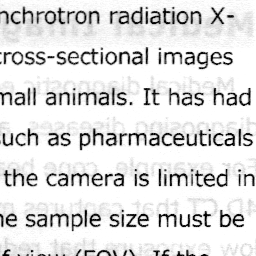

Supplement: S2 File — (ZIP) [file pone.0305208.s002.zip › S2_File/im_real1_back.bmp]

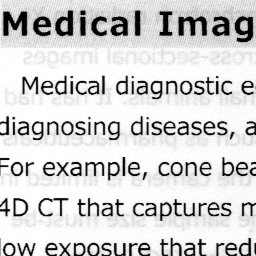

Supplement: S2 File — (ZIP) [file pone.0305208.s002.zip › S2_File/im_real1_front.bmp]

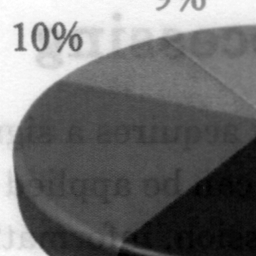

Supplement: S2 File — (ZIP) [file pone.0305208.s002.zip › S2_File/im_real2_back.bmp]

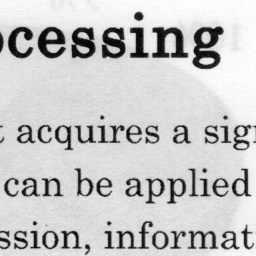

Supplement: S2 File — (ZIP) [file pone.0305208.s002.zip › S2_File/im_real2_front.bmp]

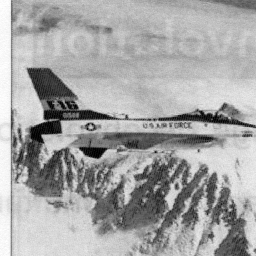

Supplement: S2 File — (ZIP) [file pone.0305208.s002.zip › S2_File/im_real3_back.bmp]

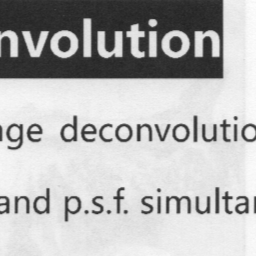

Supplement: S2 File — (ZIP) [file pone.0305208.s002.zip › S2_File/im_real3_front.bmp]
