# Supplementary material for: Show-through removal with sparsity-based blind deconvolution
Source: PLoS One. 2024 Jun 12;19(6):e0305208. doi: 10.1371/journal.pone.0305208 (PMC11168677; doi:10.1371/journal.pone.0305208)
Supplement: Attachment — Submitted filename: Response_to_the_reviewers_revision1.docx [file pone.0305208.s003.docx]

Sota Kawakami on behalf of the authors

University of Tsukuba

E-mail: kawakami.sota21@gmail.com

Phone: +818067823429

Dr. Abel C.H. Chen, Academic Editor, PLOS ONE

　　　　　　　　　　　　　　　　　　　　　　　　　　　　May 23, 2024

Dear Dr. Chen:

Thank you very much for your first review of our paper titled “Show-through removal with sparsity-based blind deconvolution”. We are grateful to the reviewers for their useful feedback that will help us improve our paper. We have carefully read the reviewer's comments and prepared a revised version. We thank you for reading the revised version of the paper. The responses to the reviewers' comments are attached to this letter. We sincerely hope that our paper will be published in PLOS ONE.

Thank you for your valuable time and effort on this paper. We look forward to hearing your decision.

Best regards,

Sota Kawakami

**Thanks to all the reviewers.**

Thank you for your comments and helpful suggestions to improve our paper. We have incorporated all your suggestions into the revised version as explained below.

**Response to the reviewer #1**

**Consern#1: The time required for the algorithm should be examined through an example.**

We added the computation times of our simulation experiments in Tables 6 and 7 (Page 17 and 18). Furthermore, Section 5.1.6 was added to explain Tables 6 and 7 (Page 17). From Table 6, it is observed that the optimization method using APG + SVP described in Section 4.2.2 significantly reduces the computation time compared with the PG + SVP. From Table 7, it is observed that the proposed method needs a longer computation time than the other comparison methods. This drawback needs to be overcome in future work, for example by using parallel processing.

**Consern#2: To enhance interpretability, the authors should specify the type of system employed for simulating the algorithm. Details such as the simulation environment (Matlab, C, Python, etc.), hardware (CPU or GPU), operating system (Windows or Linux), and other relevant information should be included in the text.**

We added Table 1 which summarizes the hardware and software environment used in the experimental studies in Section 5.1 (Page 11). Furthermore, one sentence was added to explain Table 1 (Page 11).

**Consern#3:** **The discussion of the convergence of the presented method may be lengthy and challenging in terms of theory. However, it is recommended that the authors demonstrate the convergence of the method in the numerical results section by plotting the changes in PSNR and SSIM values against the number of iterations.**

We performed new experiments (experiments 5 and 6) in Section 5.1.3 to evaluate the convergence of the proposed method with the recommended APG + SVP optimization method. We added Fig 8 which shows graphs of the changes of PSNR and SSIM indices according to the iteration number (Page 14). Furthermore, Section 5.1.3 was added to explain Fig 8 (Page 14). From Fig 8, it is observed that both PSNR and SSIM converges at 200 iterations and the convergence is stable with no divergence or oscillating behavior. From this experiment, we think that it can be said that the convergence of the method is guaranteed and 200 iterations are sufficient for the convergence.

In addition, in the experiments of the optimization methods in Section 5.1.4, we added Fig 9, which shows graphs of the changes of PSNR index for each optimization method (Page 15). Furthermore, a sentence was added to explain Fig 9 (Page 15). It is observed that the recommended APG + SVP method converged fastest among all the four investigated optimization methods.

**Consern#4:** **In order to be able to simulate the algorithm, the source of the images used in the paper must be stated.**

All images used in the simulation studies were artificially generated by ourselves. We added a sentence to state this fact in Section 5.1 (Page 11). All images used in the real image experiments were acquired by ourselves by scanning real papers having printed materials on the both sides using an electronic scanner. We added a sentence to state this fact in Section 5.2 (Page 18).

For other researchers, all the image files we used in the experiments are attached to S1 (images used in the simulation experiments) and S2 (images used in the real image experiments) in Supporting Information Section (Page 20).

**Consern#5:** **The selection of the regularization parameters (\alpha and \beta) in the total variation problems are a challenging issue and various methods have been introduced to select these parameters. In the current paper, these parameters have been selected as a fixed number. However, different results are obtained by changing these parameters. The authors are advised to calculate the PSNR and SSIM values in a table for different values of these parameters.**

The proposed method involves two regularization parameters (*i.e.* $\alpha$and $\beta$). We added Section 5.1.2 (Page 13), which explains the effect of $\alpha$ and $\beta$ on quality of the restored image. This section includes results of newly performed experiments (Figs 5, 6, 7 and Table 3) (Page 13 and 14), which demonstrate the effect of $\beta$ on the image quality. We believe that this answers to your comments in a good way. We repeat the added Section 5.1.2 in the next paragraph for your convenience.

The proposed method involves two regularization parameters (*i.e.* $\alpha$and $\beta$). This section demonstrates the effect of $\alpha$ and $\beta$ on quality of the restored image. The first parameter $\alpha$ in Eq (16) is the parameter to evaluate the magnitude of the nuclear norm. However, the proposed method with the recommended AGP + SVP optimization method does not need to adjust $\alpha$. This is because it uses the singular value projection (SVP) instead of the nuclear norm reduction, which directly imposes the constraint that the rank of $X$ is one by computing only one largest singular value and discarding other singular values. The second parameter $\beta$ affects quality of the restored image. To evaluate the effect of $\beta$ on the image quality, the same simulation experiments as in Experiment 1 and Experiment 2 were performed with five different values of *β* (*i.e.* $\beta=0.0,1.0, 2.5, 5.0, 10.0$). These experiments are named as Experiment 3 and Experiment 4, respectively. Figs 5 and 6 show zoomed small parts of the restored images for each value of $\beta$. Table 3 summarizes PSNR and SSIM indices for each value of $\beta$. Fig 7 shows changes of PSNR and SSIM indices with different $\beta$ values. It is observed that increasing $\beta$ value has no significant effect on large edge parts such as those in Figs 5(a), (b) and 6(a), but it does smooth out fine edges such as those in Fig 6(b). From Fig 7 and Table 3, it is also observed that if $\beta$ is made too large, PSNR and SSIM indices become worse and $\beta=2.5$ yields the best restoration accuracy in average for the setup of Experiments 3 and 4. Finally, we mention that the image quality was not very sensitive to $\beta$ value than what we expected before these experiments.

**Response to the reviewer #2:** **Why you didn't use autoencoder as anomaly detection? Autoencoders is a good self-supervised machine learning technique which can use as anomaly detection (Autoencoders and their Applications inMachine Learning: A Survey, DOI: 10.1007/s10462-023-10662-6).**

After reading your comments, we performed a literature survey. Consequently, we found several show-through removal methods using Auto-encoder. For example, these include a supervised method (DOI:10.1016/j.patcog.2018.08.011), a self-supervised method (DOI:10.48550/arXiv.2203.04814), and an unsupervised method (DOI:10.1016/j.patcog.2021.108099). All of them are based on binarization. We added the most famous method (DOI:10.1016/j.patcog.2018.08.011) as a reference [14] in Introduction (Page 3). Furthermore, in Introduction, we added our short comment repeated in the next paragraphs on the comparison between the optimization-based approach and the Auto-encoder-based approach (Page 3).

Also, in recent years, a show-through removal method using Convolutional Neural Network (CNN) [13] has also been proposed. Among them, the method based on image binarization using Auto-encoder [14] is promising. The Auto-encoder-based approach like [14] can certainly perform accurate show-through removal with only one-sided input image. However, it has the following drawbacks. Since the method uses the image binarization, images with gradations or shadings cannot be recovered well. Furthermore, their accuracy depends on used training data. On the other hand, our proposed method requires both of an image of the fore side and that of the back side, but can be applied to images with gradations or shadings. At the current stage, we believe that both the optimization-based approach like ours and the Auto-encoder-based approach are meaningful to study, because they have different advantages and disadvantages and the both may be able to be combined in the future to develop more accurate or more convenient methods.

**Other points**

We corrected the following careless mistakes in the previous version.

1. Corrected spelling errors in Section 4.2.1.
2. Changed the SSIM values in Tables 2 and 5 (corresponding to Tables 1 and 3 in the previous version). The reason is that we found a bug in the SSIM calculation code and recalculated the SSIM values after fixing the bug. This modification did not significantly affect the results of the experiments.
3. The cropped images in Figs 13 and 14 (corresponding to Figs 8 and 9 in the previous version) were incorrect and have been changed to the correct ones.

We modified the following points to satisfy PLOS ONE's style requirement.

1. Added captions for each figure in the manuscript.
2. The legend caption in the image was removed.
3. The resolution of the images was changed to acceptable one.
4. Add page number.
5. The code implementing this method was uploaded to “https://zenodo.org/records/1101626”.
