# Supplementary material for: Show-through removal with sparsity-based blind deconvolution
Source: PLoS One. 2024 Jun 12;19(6):e0305208. doi: 10.1371/journal.pone.0305208 (PMC11168677; doi:10.1371/journal.pone.0305208)
Supplement: Attachment — Submitted filename: Response_to_the_reviewers_revision2.docx [file pone.0305208.s004.docx]

Sota Kawakami on behalf of the authors

University of Tsukuba

E-mail: kawakami.sota21@gmail.com

Phone: +818067823429

Dr. Abel C.H. Chen, Academic Editor, PLOS ONE

　　　　　　　　　　　　　　　　　　　　　　　　　　　　May 13, 2024

Dear Dr. Chen:

Thank you very much for your second review of our paper titled “Show-through removal with sparsity-based blind deconvolution”. We are grateful to the reviewers for their useful feedback that will help us improve our paper. We have carefully read the reviewer's comments and prepared a revised version. We thank you for reading the revised version of the paper. The responses to the reviewers' comments are attached to this letter. We sincerely hope that our paper will be published in PLOS ONE.

Thank you for your valuable time and effort on this paper again. We look forward to hearing your decision.

Best regards,

Sota Kawakami

**Thanks to all the reviewers.**

Thank you for your second comments and helpful suggestions to improve our paper. We have incorporated all your suggestions into the revised version as explained below.

**Reviewer #1: After reviewing the original text, the revised versions, and the opinions of the referees, as well as considering the answers provided and evaluating the article from a scientific standpoint, I believe that the manuscript in its current form may be considered for the next phase of the editorial process. Also, as a recommendation for authors, it is suggested that the images and codes used for this article be placed on public repositories like GitHub and its address is mentioned in the text of the article. Additionally, the open-sourcing of the code not only facilitates the dissemination of the paper but also boosts its citation count.**

Thank you for your comment. According to your advice, I have added the link to repository to get images and codes used in the experiment in section 5.1 (Page 12).

**Reviewer #2: All previously provided feedback has been duly addressed. However, it's recommended to consider referencing the most recent work on autoencoders, such as "Autoencoders and their applications in machine learning: a survey (2024)," instead of "A selectional auto-encoder approach for document image binarization (2019)," to ensure the inclusion of up-to-date and comprehensive literature in the manuscript.**

Thank you for your comment. As you pointed, our reference to the approach using Auto-encoder was not good in the following sense. We cited a paper on the binarization of document image using Auto-encoder, but this is not a standard usage of Auto-encoder. Since a major application of Auto-encoder is to remove disturbance from images, which has been already proposed in image denoising, speckle reduction in SAR or ultrasound images, and clutter rejection in radar images. The same method can be also used to remove show-through from scanned images. Since the proposed optimization-based method and the Auto-encoder method have different advantages and disadvantages, it would be interesting to compare the two methods in terms of various points of views. So, according to your suggestion, we have made the following changes in the 2-nd revised version. First, we changed the cited paper (Ref. 14) from the previous one to the following new one, which you have introduced to us.

- Berahmand, K., Daneshfar, F., Salehi, E.S. et al. Autoencoders and their applications in machine learning: a survey. Artif Intell Rev 57, 28 (2024). https://doi.org/10.1007/s10462-023-10662-6

Second, we have changed the explanation on the use of Auto-encoder (on Page 3) as summarized in the next paragraph.

Also, in recent years, show-through removal methods using Convolutional Neural Network (CNN) have been proposed [13,14]. Among them, the method based on Auto-encoder [14] has a large potential. A typical major application of Auto-encoder is to remove disturbance from signals or images, which has been already proposed in image denoising, speckle reduction in SAR or ultrasound images, and clutter rejection in radar images. The same method can be also used to remove show-through from scanned images. This method requires only a single image of front side. However, its accuracy depends on used training data, and it may be weaker to the show-through having strong correlation with the front page image. On the other hand, our proposed method requires both of an image of the fore side and that of the back side, but using the two images eliminates the necessity of training data and may lead to an improved accuracy (for example, in the above mentioned case). At the current stage, we believe that both the optimization-based approach like ours and the Auto-encoder-based approach are meaningful to study, because they have different advantages and disadvantages and the both may be able to be combined in the future to develop more accurate or more convenient methods.
